# Region Specific Up-Regulation of Oxytocin Receptors in the Opioid *Oprm1*^−/−^ Mouse Model of Autism

**DOI:** 10.3389/fped.2014.00091

**Published:** 2014-09-01

**Authors:** Valentina Gigliucci, Marianna Leonzino, Marta Busnelli, Alessandra Luchetti, Viola Stella Palladino, Francesca R. D’Amato, Bice Chini

**Affiliations:** ^1^Institute of Neuroscience, National Research Council, Milan, Italy; ^2^Dipartimento di Biotecnologie e Medicina Traslazionale, Università degli Studi di Milano, Milan, Italy; ^3^Institute of Cellular Biology and Neurobiology, National Research Council, Rome, Italy; ^4^IRCCS Santa Lucia Foundation, Rome, Italy

**Keywords:** oxytocin receptor, μ-opioid receptor, brain autoradiography, social behavior, autism, *Oprm1*^−/−^ mice, ultrasonic vocalizations

## Abstract

Autism spectrum disorders (ASDs) are characterized by impaired communication, social impairments, and restricted and repetitive behaviors and interests. Recently, altered motivation and reward processes have been suggested to participate in the physiopathology of ASDs, and μ-opioid receptors (MORs) have been investigated in relation to social reward due to their involvement in the neural circuitry of reward. Mice lacking a functional MOR gene (*Oprm1*^−/−^ mice) display abnormal social behavior and major autistic-like core symptoms, making them an animal model of autism. The oxytocin (OXT) system is a key regulator of social behavior and co-operates with the opioidergic system in the modulation of social behavior. To better understand the opioid-OXT interplay in the central nervous system, we first determined the expression of the oxytocin receptor (OXTR) in the brain of WT C57BL6/J mice by quantitative autoradiography; we then evaluated OXTR regional alterations in *Oprm1*^−/−^ mice. Moreover, we tested these mice in a paradigm of social behavior, the male–female social interaction test, and analyzed the effects of acute intranasal OXT treatment on their performance. In autoradiography, *Oprm1*^−/−^ mice selectively displayed increased OXTR expression in the Medial Anterior Olfactory Nucleus, the Central and Medial Amygdaloid nuclei, and the Nucleus Accumbens. Our behavioral results confirmed that *Oprm1*^−/−^ male mice displayed social impairments, as indicated by reduced ultrasonic calls, and that these were rescued by a single intranasal administration of OXT. Taken together, our results provide evidence of an interaction between OXT and opioids in socially relevant brain areas and in the modulation of social behavior. Moreover, they suggest that the oxytocinergic system may act as a compensative mechanism to bypass and/or restore alterations in circuits linked to impaired social behavior.

## Introduction

Autism spectrum disorders (ASDs) are characterized by a triad of symptoms that includes impaired communication, social impairments, and restricted and repetitive behaviors and interests (1). The prevailing hypothesis regarding the physiopathology of ASDs identifies the area of social cognition as a primary deficit. In particular, it focuses on the impaired capabilities of affected people to attribute mental states to others (and oneself) in order to explain and predict behavior (i.e., the theory of mind hypothesis proposed by Baron-Cohen and colleagues (2, 3). However, altered affectivity is evident in autistic children (4) and, as recently proposed by Chevallier and others (5), motivation and reward processes might also participate to the physiopathology of ASDs. Reward circuitry dysfunctions might lead to deficits in social seeking and maintenance, resulting in reduced social capabilities and interests, and if appearing early in life, in social learning. Deficit in social cognition will thus be a consequence, rather than a cause, of impaired social behavior. Excessive brain opiate activity has been proposed in the past as a neurochemical feature in autism (6) and due to their involvement in the neural circuitry of reward, μ-opioid receptors (MORs) have also been investigated in relation to social reward, emotion, and social behavior (7–9), and represent a key target to understand the neurobiological basis of social reward dysfunction in humans and animals.

In humans, MOR activation in specific brain regions such as the amygdala, the periaqueductal gray, and the subgenual cingulated cortex is believed to be protective or adaptive in relation to social rejection. In fact, positron emission tomography (PET) scanning showed that social rejection, exemplified by a paradigm in which the test subject shows interest toward another individual who does not return it, could increase the binding of endogenous opioids to MOR in these areas, where greater binding also seemed to correlate to better resiliency. In a contest of social acceptance, MOR activation in the ventral striatum was shown to correlate with desire for social interaction (10). In a similar study, the G variant of the A118G polymorphism of the MOR-encoding gene (OPRM1) correlated with a greater sensitivity to social rejection, reflected in a higher activation of specific brain regions, such as the dorsal anterior cingulate cortex and the anterior insula. Genetic variations in the OPRM1 gene can also influence individuals’ ability to engage in social interactions. People carrying the G allele of the A118G polymorphism of the OPRM1 gene seem to experience a greater pleasure during social situations and they tend to engage in affectionate relationships more easily in comparison with people carrying the more common A variant of the gene (11). A crucial role of MOR in partner preference has also been established by studies in prairie and mountain voles. In the monogamous prairie vole, dorsal striatal MOR has been proved fundamental for the development of partner preference, which leads to the establishment of pair bonding, although pharmacological disruption of MOR signaling did not consistently alter the pattern of mating behaviors (9, 12).

In species where individuals develop selective affective bonds during their life, such as sheep or primates, MOR signaling has been implicated in the modulation of the mother–infant attachment: in infant rhesus macaques the genetic variant C77G of the MOR gene, which increases its affinity for β-endorphin (13), has been associated with increased attachment to the mother and stronger protest response and distress during separation (14). At the same time, the maternal attachment toward the offspring seems to be subjected to MOR effects. Higham and colleagues (15) evidenced that free-ranging macaque females carrying at least one copy of the minor allele (G) of the OPRM1 gene were more possessive toward their infants than mothers homozygous for the C allele. Mouse pups where the MOR gene (*Oprm1)* has been permanently disrupted (*Oprm1*^−/−^ mice) produce fewer ultrasound vocalizations (USVs) in response to isolation from the mother when compared to wild type mice, to indicate that lack of MOR may induce resilience to isolation (16). Moreover, it is possible that the lack of MOR prevents the establishment of an association between maternal stimuli and feelings of reward, as transgenic mice do not show a marked preference for a familiar environment over an unfamiliar one (16). Later on in their life, these mice display reduced interest for interaction with other mice of the same sex and age (17) and they appear indifferent to ultrasounds emitted by mice of the opposite sex (18). A recent extensive behavioral characterization of *Oprm1*^−/−^ mice confirmed that these animals display major autistic-like core symptoms and elegantly provided key neuroanatomical and neurofunctional correlates (19). In particular, *Oprm1*^−/−^ mice display abnormal social behavior, as evidenced by a decreased time in close social contact and increased self-grooming in the direct social interaction test, reduced sociability, and social novelty recognition in the three chamber test, accompanied by increased aggression, and impaired ability in building a nest. These mice also display increased perseverative and stereotyped behaviors such as increased rearing, grooming, circling, and head shaking associated with behavioral inflexibility, as evidenced by deficient pattern of exploration in a Y-maze test. Concerning the neurobiological substrates of such phenotype, of particular interest is the observation of changes in oxytocin (OXT) gene expression in specific areas of the brain: OXT transcripts were found to be reduced in the Nucleus accumbens (NAcc) but not in the Caudate–putamen (CPu) and Central amygdala (CeA) (19).

Together with the opioid system, the OXT system is a key regulator of all the aspects of social behavior, including those involved in reproduction and care of the offspring (20). In humans, OXT facilitates the processing of social information, improves cognitive emphatic abilities and increases interpersonal trust (21). As originally put forward by Modahl (22), a deficit in the OXT system linked to an altered opioid regulation may underline the social deficits in autism. Evidence of opioid–OXT interactions is indeed well established. Endogenous opioids are involved in the modulation of OXT release into the brain and the periphery via mu- and kappa-receptors expressed on OXT-secreting neurons (23–26). Even though the opioidergic modulation of OXT release may account for many shared effects, the interplay of these two systems does not probably end with that. A social motivation circuitry in which OXT, vasopressin, endogenous opioids, and catecholamines were hypothesized to participate in a wide variety of affiliative behaviors was proposed more than 15 years ago (27) and has been more recently integrated into a network of neurobiological mechanisms, which include neuronal, neurotransmitters, and hormone systems whose alterations could underline the social impairment observed in autism (28).

To contribute to unravel the critical interactions between the opioid and OXT systems in the brain, we first reviewed the literature on oxytocin receptor (OXTR) and MOR distributions in the mouse brain. As shown in Table 1, we found an overlapped receptors’ expression in several regions involved in social behavior.

**Table 1:** **MOR and OXTR expression levels in mouse brain as reported in the literature**.

Brain region	MOR	Reference	OXTR	Reference
Olfactory bulb	+	(29–31)	+++	(32)
Anterior olfactory nucleus	++	(31)	+++	(32 –34)
Lateral Septum	+/++	(29–31)	+++	(32, 34)
Bed nucleus of the stria terminalis	++	(29–31)	+/+++	(32)
Amygdala
Basolateral (comprising BLA and BLP)	++/+	(29–31)	++	(33)
Medial	++++	(29–31)	++	(32)
Central	++++	(31)	++/+++	(32, 33)
Cortical amygdaloid area	++	(31)	+++/++++	(32, 33)
Amygdalohippocampal area	+	(31)	++/+++	(33)
Hippocampus	+	(29–31)	++	(32–34)
Caudate–putamen	++/+++	(29–31)	+	(32)
Nucleus accumbens	++++/++	(29–31)	+/++++	(32–34)
Paraventricular thalamic nucleus	+++	(31)	++	(32, 33)
Habenula	++++	(29–31)	N.D.	

This observation suggests that OXTR and MOR may reciprocally modulate each other even at the cellular and/or molecular level. To investigate, if alterations in MOR expression might induce changes in the OXTergic system we decided to evaluate the expression and distribution of OXTRs in the brain of *Oprm1*^−/−^ mice.

Oxytocin receptors represent the pharmacological target of OXT, and OXT administration has been proposed as a potential treatment of social deficits in autistic patients (35). In particular, intranasal OXT administration is believed to circumvent the poor blood–brain barrier (BBB) permeability of this peptide. Even if the direct passage of intranasal OXT into the brain is still matter of debate (36, 37) acute and chronic intranasal OXT administration have been shown to exert behavioral effects in rodents (34, 38, 39). Even if it cannot be excluded that some of the behavioral effects of OXT are mediated via peripheral mechanisms, intranasal OXT administration in awake animals represents at present the most convenient and reproducible method to assess the therapeutic effects of this peptide on social behavior. We thus tested *Oprm1*^−/−^ mice in a paradigm of social behavior and analyzed the effect of intranasal OXT treatment on their behavioral performances.

## Materials and Methods

### Animals and housing conditions

*Oprm1*^+/+^ (WT) and *Oprm1*^−/−^ mice were used in this study. *Oprm1*^−/−^ mice were generated by disruption of exon 2 in the *Oprm1* gene as described elsewhere (40). The homozygotic parents (*Oprm1*^+/+^ and *Oprm1*^−/−^) were derived from heterozygous breeding pairs that were fully backcrossed on a C57BL6/J genetic background. The two homozygous lines were maintained separately. Animals were weaned when 28-day-old and maintained in same sex/genotype groups of four to five subjects in transparent high-temperature polysulfone cages (27 cm × 21 cm × 14 cm) with water and food available *ad libitum* (2018 Teklad Global 18% Protein Rodent Diet, Harlan, Lyon, France). Room temperature (21 ± 1°C) and a 12:12 h light–dark cycle (lights on at 1900 h) were kept constant.

Two different groups of adult WT and *Oprm1*^−/−^ male mice (3–4 months old) were used: the first group (4 WT and 3 *Oprm1*^−/−^ mice, one subject per litter) was used for autoradiography and histological examination; the second group of males (18 WT from 6 litters and 17 *Oprm1*^−/−^ mice from 7 litters) underwent intranasal OXT treatment; USVs and behavior during exposure to a female partner were observed shortly after. The genotype of all animals used in this study was controlled by PCR at the end of the experiment, according to already described procedures (40). Every animal procedure used was in strict accordance with standard ethical guidelines (European Community Guidelines on the Care and Use of Laboratory Animals 2010/63/EU) and the Italian legislation on animal experimentation (D.Lvo 116/92).

### OXTR autoradiography

Naïve WT and *Oprm1*^−/−^ mice were sacrificed by cervical dislocation, the brains quickly removed and immediately frozen by immersion in cold isopentane at −25°C and subsequent storage at −80°C.

Coronal brain sections (14 μm) were sliced with a cryostat, thaw-mounted on microscope slides pre-coated with chrome-alum–gelatin and kept at −80°C until further use.

Oxytocin receptor autoradiography was performed as described in Huang et al. (34). Briefly, sections were fixed with 0.2% paraformaldehyde in 0.1 M phosphate-buffered saline (pH 7.4) and rinsed twice with 0.1% bovine serum albumin in 50 mM Tris-HCl buffer (pH 7.4). OXT binding sites were detected by incubation (1 h at room temperature in a humid chamber) with the radioiodinated OXTR antagonist ornithine vasotocin analog ([^125^I]-OVTA, specific activity 2200 Ci/mmol; Perkin Elmer, MA, USA) at 0.02 nM in a medium containing 50 mM Tris-HCl, 0.025% bacitracin, 5 mM MgCl_2_, and 0.1% bovine serum albumin. Sections immediately adjacent to the ones used for [^125^I]-OVTA binding were used to determine non-specific binding by addition of 2 μM OXT to the incubation solution.

At the end of binding, the unbound excess of ligand was washed out by two rinses in ice-cold incubation medium and a final rinse in cold distilled water. The slides were quickly dried under a stream of cool air and exposed to Biomax MR Films (Kodak) in an autoradiographic cassette for 72 h. The final autoradiograms were digitalized by grayscale high-resolution scanning (600 × 600 dpi) and analysis of the optical binding density of the brain regions of interest (ROIs) was carried out using the ImageJ 1.47v software (NIH, USA). ROIs were identified by comparison with a reference mouse brain atlas (41) and manually delineated with the ROI manager tool of the software. Specific densitometric gray intensity was calculated by subtraction of the gray level of the respective section treated for non-specific binding. For each animal, the final gray intensities of each brain region were calculated by averaging two [for anterior olfactory nucleus (AON), LS, AHiPM, BLP, and PMCo] or three (for OB, NAcc, CPu, Hipp CA3, PV, Hb, BLA, MeA, and CeA) sections at different coronal planes from bregma. The regions of a limited rostro-caudal extension [medial AON (AONm) and BNST] were analyzed on a single coronal plane from bregma. Brain regions were selected on the basis of co-expression of OXTR and MOR receptors at medium-high level as resulting from a review of the data currently available in the literature and summarized in Table 1. Even though in literature neither OXTR nor MOR expressions in the hippocampus are reported to be high, we also included the CA3 field of this region in our analysis because it appeared intensely labeled in WT mice. Binding specificity was ensured by comparison with the adjacent sections incubated with an excess of OXT in order to displace any OVTA specifically bound to the OXTRs.

Autoradiographic ^125^I microscales (Amersham International, UK) also were exposed for 72 h and a reference standard curve was generated. Levels of gray intensity were then converted to nanocurie per milligram tissue equivalent by interpolation with the standard curve.

In order to increase accuracy in identifying the brain regions within each brain section, the slides labeled for non-specific binding and the slides labeled for total binding were further colored with Nissl staining and acetylcholinesterase (AChE) staining, respectively (see below).

### Histological staining

#### Nissl staining

The non-specific binding labeled slides were treated for Nissl staining. They were defatted by immersion in deionized water (2 min) followed by subsequent immersions in increasing concentrations of ethanol (EtOH 70% v/v, 95% v/v, and 100%, 2 min each). Sections were then rehydrated by 30 s immersions in decreasing concentrations of ethanol (EtOH 100%, 95% v/v, and 70% v/v) and after a final dip in deionized water they were left for 6 h in cresyl violet solution (0.1% cresyl violet, 0.65% sodium acetate trihydrate in 0.5% acetic acid, pH 3.3). Differentiation was obtained by two consecutive changes (3 s each) of deionized water, EtOH 70% (v/v) and EtOH 95% (v/v), and final dehydration was achieved by two changes (10 s each) in EtOH 100%. Sections were finally cleared by immersion in xylene and coverslips were mounted onto the slides with permanent mounting medium (Entellan^®^, Merck-Millipore, Germany) and left to dry overnight under a fume hood.

#### Acetylcholinesterase staining

The slides labeled for total binding in autoradiography were processed for AChE staining following the protocol described by Franklin and Paxinos (41). All the reagents used in this procedure were obtained from Sigma Aldrich (Italy) with the exception of the mounting medium. Sections were immersed over night at room temperature in an incubation solution (50 mM sodium acetate, 4 mM copper sulfate, 16 mM glycine, 4 mM S-acetylthiocholine iodide, 10 nM ethopropazine, and pH 5.0 with HCl 1 N) and developed the following day by incubation for 10 min at room temperature in a solution containing 1% sodium sulfide (pH 7.5 with glacial acetic acid). The colored precipitate from the reaction was fixed to the sections by an overnight incubation with formalin 10%. Finally, the slides were left to dry under a fume hood, dehydrated by subsequent immersions in ethanol 100% and xylene 100%, coverslipped with permanent mounting medium (Entellan^®^, Merck-Millipore, Germany) and left to dry overnight under a fume hood.

For both histological protocols, dried slides were digitalized at high resolution and the obtained images were used as guidance for the identification of brain regions within the sections.

### Behavioral effects of OXT intranasal administration

#### Oxytocin intranasal administration

Adult males were gently handled during the 4 days before testing to progressively habituate to the intranasal administration protocol. The first-day they were simply handled, the second-day they were firmly kept, the third-day they were firmly kept in supine position with their back supported by the palm of the manipulator’s hand, and the fourth-day a drop of saline was introduced in each nostril. After the last manipulation, they were isolated in clean cages for 24 h, treated with OXT or saline and their vocalizations and behavior were recorded.

Oxytocin (Sigma Aldrich, Italy) was dissolved in saline (0.9% NaCl) to a concentration of 0.6 mg/10 ml. A total volume of 5 μl of the OXT solution was administered intranasally by gently placing drops in each nostril, that were taken in when the mice reflexively inhaled (600 ng OXT/mouse). The dosage of OXT was based on data from the literature (34, 38, 39). Control mice received an equal volume of saline (Veh). A 20-μl Eppendorf pipette with gel-loading tips was used for administration. Administration was rapid (<15 s) and handling was consistent across treatment groups.

#### Male–female social interaction test

Adult WT and *Oprm1*^−/−^ males were isolated for 24 h in clean cages. A total of 17 *Oprm1*^−/−^ (8 OXT and 9 Veh) and 18 WT (9 OXT and 9 Veh) males was used as subjects and an equivalent number of females of the two genotypes were used as partners. Each animal was used only once and administered with OXT or saline. The estrous cycle of the female partners was not considered, as irrelevant on USV quantitative performance during the first minutes of interaction in sexual naïve males facing unfamiliar females (42–44). Five minutes after the male’s intranasal drug/saline administration, a female of the same genotype was introduced into the male’s home cage and left for 5 min. The delay of only 5 min was chosen according to previous studies analyzing the behavioral effects of OXT after intranasal administration (34, 39). *Oprm1*^−/−^ males were exposed to *Oprm1*^−/−^ females and WT males to WT females. The cage was placed in front of a video-camera and under a microphone for the recording and subsequent analysis of behavior and ultrasonic vocalizations. The videos were analyzed with the Observer software (Noldus Technology, The Netherlands) and the following males’ behaviors were considered for the statistical analysis: locomotion, exploration (sniffing, rearing, digging, and climbing), social investigation (following, sniffing nose, body, and ano-genital region of the partner), and self-grooming. Ultrasonic vocalizations were recorded using an UltraSoundGate Condenser Microphone (CM16, Avisoft Bioacoustics, Berlin, Germany) lowered 5 cm above the top of the cage, and analyzed by SasLab Pro (version 4.40; Avisoft Bioacoustics). Details concerning USVs recording and analysis can be found in previous papers (17, 45). According to previous literature, USVs recorded during the first minutes of male–female social interaction, are considered to be mainly uttered by the male (46).

### Statistical analysis

In autoradiography, we quantified the intensity of the binding signal in *Oprm1*^−/−^ mice and compared it with the binding showed by WT mice. Average nanocurie per milligram of tissue equivalents were compared by Student’s *t*-test with GraphPad Prism 5.0 (GraphPad Software, USA). Results were deemed statistically significant when *p* < 0.05. The effect of the genotype and treatment on behavioral measures was first analyzed by multiple analysis of variance (MANOVA) followed by univariate ANOVAs and, in cases of significance (*p* < 0.05), Tukey HSD *post hoc* tests.

## Results

### Quantitative analysis of OXTR distribution in WT mice brain

**Figure 1:**
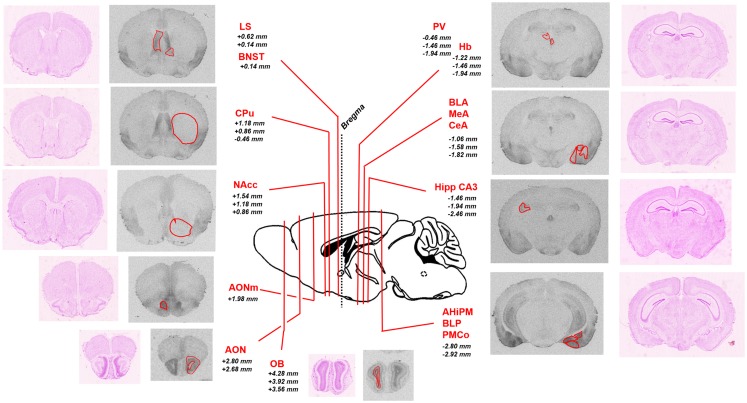
**Schematic representation of the brain regions of interest analyzed in the study**. Representative sections of autoradiographic labeling of OXTR with [^125^I]-OVTA 20 pM are displayed in grayscale, flanked by corresponding Nissl stained sections. Red contours outline the regions of interest analyzed. A sagittal schematic representation of a mouse brain is reported in the central panel. Lines indicate the antero-posterior localization of the coronal sections taken into account, defined by the reported distances (millimeter) from bregma (according to the Franklin and Paxinos (41) mouse brain atlas). AHiPM, amygdalohippocampal area, posteromedial part; AON, anterior olfactory nucleus; AONm, anterior olfactory nucleus, medial part; BLA, basolateral amygdaloid nucleus, anterior part; BLP, basolateral amygdaloid nucleus, posterior part; BNST, bed nucleus of the stria terminalis; CeA, central amygdaloid nucleus; CPu, caudate–putamen; Hb, habenular nucleus; Hipp CA3, hippocampus, field CA3; LS, lateral septum; MeA, medial amygdaloid nucleus; NAcc, nucleus accumbens; OB, olfactory bulb; PMCo, posteromedial cortical amygdaloid area.

To analyze the pattern of OXTR distribution in *Oprm1*^−/−^ mice, we first analyzed [^125^I]-OVTA binding data in WT mice on which *Oprm1*^−/−^ animals are backcrossed. Figure 1 shows representative autoradiographic sections for the selected area and associated Nissl staining of adjacent slices; the reported distances from bregma of the different coronal planes are deduced from a reference mouse brain atlas (41).

**Figure 2:**
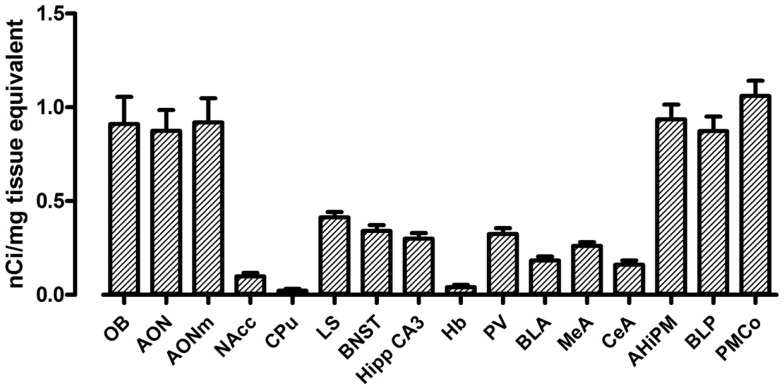
**Regional distribution and quantification of OXTR in WT mice**. Average regional expression of OXTR defined by [^125^I]-OVTA binding in WT mice. For each animal, final levels of OXTR expression were calculated averaging data obtained from the different coronal planes analyzed within the same region of interest. Each bar represents data expressed as mean and SEM of four animals.

The use of a commercial ^125^I microscales standard allowed us to quantify the levels of [^125^I]-OVTA binding in the brain of our mice. Quantification of OXTR expression in several areas of the brain in WT mice is reported in Figure 2. In WT mice, we found the highest levels of OXTR (here in the text reported as mean nanocurie per milligram tissue equivalent ± SD) in the olfactory bulb (OB, 0.91 ± 0.25), the AON (0.88 ± 0.22), and in some posterior nuclei of the amygdala, specifically the amygdalohippocampal area (AHiPM, 0.94 ± 0.16), the posteromedial cortical amygdaloid area (PMCo, 1.06 ± 0.16), and the posterior part of the basolateral amygdaloid nucleus (BLP, 0.87 ± 0.16). Medium level of OVTA binding was detected in lateral septum (LS, 0.41 ± 0.06), the bed nucleus of the stria terminalis (BNST, 0.34 ± 0.06), the CA3 field of the hippocampus (Hipp CA3, 0.30 ± 0.06), the paraventricular thalamic nucleus (PV, 0.32 ± 0.06), and in the medial amygdaloid nucleus (MeA, 0.26 ± 0.04). In the other anterior nuclei of the amygdala analyzed, the CeA nucleus (0.16 ± 0.05) and the anterior part of the basolateral amygdaloid nucleus (BLA, 0.18 ± 0.04), and in the NAcc (0.10 ± 0.04) OXTR expression was rather low. Interestingly, Dölen et al. (33) report the basolateral nucleus of the amygdala as a whole to display “average” OXTR expression, however, from observation during analysis we noticed a clear difference in OXTR expression between the anterior and the posterior parts of this nucleus, therefore, we deemed it more appropriate to analyze the two subregions separately, distinguishing them into BLA and BLP. Finally, we found minimal expression of OXTR in the CPu (0.02 ± 0.02) and the habenula (Hb, 0.04 ± 0.02) of WT mice. Overall, the expression and the distribution of OXTR in the brains of WT mice, at least in relation to the regions analyzed, is consistent with what has been previously reported (see Table 1).

The regions with the lowest OXTR binding signal (CPu and Hb) presented a 20-fold lower signal compared to the regions with the highest OXTR expression such as the PMCo. The NAcc also showed a very low level of OXTR expression, being it around a tenth of the PMCo’s OXTR expression. All the remaining regions, such as the anterior nuclei of the amygdala, the LS, the BNST, and the PV, showed instead an average OXTR expression, comprised between one-fifth and one-half of the PMCo’s levels.

### Absence of MOR induces specific increases of OXTR expression in the AONm, in the anterior amygdaloid nuclei and in NAccs

In order to reveal possible interaction(s) between MOR and OXTR, we looked for alterations in oxytocinergic binding levels in the brains of *Oprm1*^−/−^ mice.

**Figure 3:**
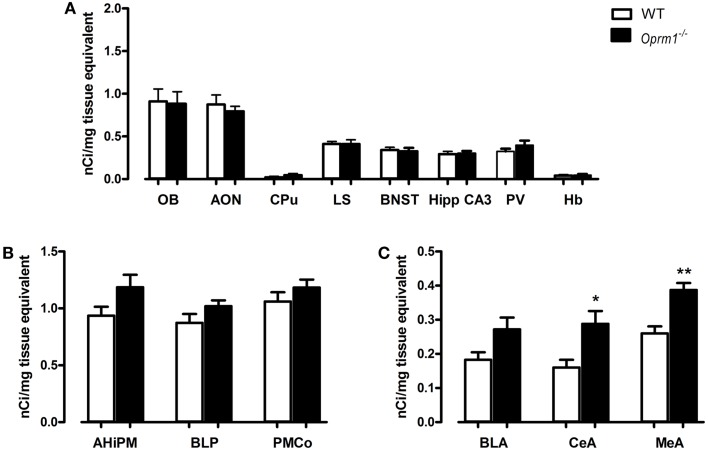
***Oprm1*^−/−^ mice display increased OXTR expression selectively in the anterior nuclei of the amygdala**. Average regional expression of OXTR defined by [^125^I]-OVTA binding in *Oprm1*^−/−^ mice in comparison with WT mice. **(A)** No differences in OXTR expression were detected in *Oprm1*^−/−^ mice in several brain regions reported to co-express medium-high levels of MOR and OXTR. **(B)** OXTR expression is not influenced by the lack of MOR in the posterior nuclei of the amygdala whereas **(C)** an increase of OXTR is detectable in the anterior nuclei of the amygdala CeA and MeA. Data expressed as mean and SEM of three to four animals. *p < 0.05, **p < 0.01 compared to WT controls.

Our data indicate that the expression of OXTR did not differ significantly between WT and *Oprm1*^−/−^ mice in the OB {*t*-test; [*t*(_4_) = 0.139, *p* = 0.897, n.s.]}, AON {coronal planes corresponding to its central part; *t*-test; [*t*(_5_) = 0.577, *p* = 0.589, n.s.]}, CPu {*t*-test; [*t*(_4_) = 1.238, *p* = 0.284, n.s.]}, LS {*t*-test; [*t*(_5_) = 0.046, *p* = 0.965, n.s.]}, BNST {*t*-test; [*t*(_5_) = 0.315, *p* = 0.766, n.s.]}, Hipp CA3 {*t*-test; [*t*(_5_) = 0.160, *p* = 0.880, n.s.]}, PV {*t*-test; [*t*(_5_) = 1.269, *p* = 0.260, n.s.]}, and Hb {*t*-test; [*t*(_5_) = 0.219, *p* = 0.835, n.s.]} (Figure 3A). Similarly, the posterior nuclei of the amygdala (AHiPM, BLP, and PMCo), did not present alterations in OXTR expression linked to the lack of MOR {*t*-test; [*t*(_5_) = 1.891, *p* = 0.117, n.s.], [*t*(_5_) = 1.442, *p* = 0.209, n.s.], and [*t*(_5_) = 1.087, *p* = 0.327, n.s.], respectively} (Figure 3B).

On the contrary, in the anterior nuclei of the amygdala, and in particularly in the CeA and MeA, the absence of MOR led to an up-regulation of OXTR {*t*-test; [*t*(_5_) = 3.102, *p* < 0.05] and [*t*(_5_) = 4.299, *p* < 0.01], respectively, Figure 3C}. A trend (*p* = 0.07) toward an increase in OXTR was also found in the anterior basolateral amygdala (Figure 3C).

**Figure 4:**
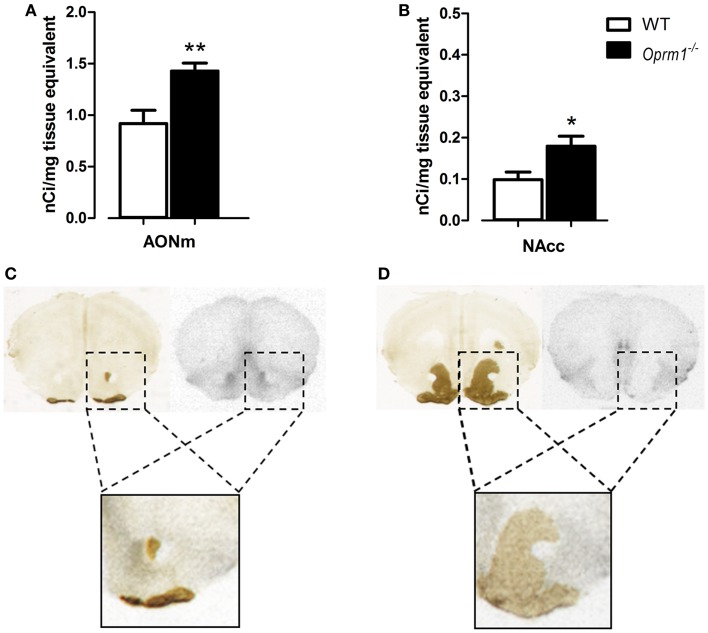
***Oprm1*^−/−^ mice display increased OXTR expression in the medial AON and the nucleus accumbens**. Average regional expression of OXTR defined by [^125^I]-OVTA binding in *Oprm1*^−/−^ mice in comparison with WT mice. OXTR expression is increased in both **(A)** AONm and **(B)** NAcc of *Oprm1*^−/−^ mice. At the zone of transition between AON and NAcc, distinction between the two structures was determined by AChE staining, which selectively labels the NAcc and the olfactory tubercle. **(C)** The spot of intense [^125^I]-OVTA labeling evident in the autoradiographic picture and the AChE staining, apparently in the same position, do not overlap when the two images are merged (blow-up), indicating that the NAcc doesn’t express high levels of OXTR in this area. **(D)** Superimposition of the autoradiogram and the AChE staining of a coronal section of the NAcc where the AONm is not present anymore (blow-up) confirms that this region does not express high levels of OXTR.

Another region in which we found an up-regulation of OXTR is the AONm in the posterior part of AON {*t*-test; [*t*(_7_) = 3.590, *p* < 0.01]} (Figure 4A). Because discriminating between the AON and NAcc is particularly tricky in this area of the brain, we performed AChE staining on the same slices processed for autoradiography. AChE selectively labels the NAcc and the olfactory tubercle but not the AON, thus allowing unambiguous identification of the two structures. Figures 4C,D show examples of brain sections labeled with [^125^I]-OVTA and AChE. In the blow-ups of the merged images it is evident that the strong autoradiographic signal observed in this area does not overlap with the AChE staining, but it is adjacent to it. This allowed us to assign the strong specific [^125^I]-OVTA labeling of this region to the AONm. Given the difficulty, in this region, to discriminate between AONm and NAcc in absence of AChE staining, we cannot exclude that the area identified as NAcc in (34) may, instead, correspond to the AONm.

Finally, in the NAcc of *Oprm1*^−/−^ mice, sampled at three different coronal planes, we found an OXTR level twice as high as that observed in WT animals {*t*-test; [*t*(_5_) = 2.767, *p* < 0.05]} (Figure 4B), suggesting that the NAcc is an anatomical field of compensative interaction(s) between the oxytocinergic and the opioidergic systems.

### Behavioral effects of intranasal administration of oxytocin in *Oprm1*^−/−^ mice

**Figure 5:**
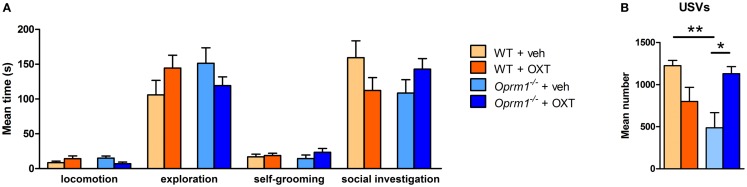
**Acute intranasal administration of OXT rescues the impairments in ultrasound emission in *Oprm1*^−/−^ mice during male–female social interaction**. Effects of acute intranasal OXT administration (600 ng) in *Oprm1*^−/−^ and WT male mice during a 5 min male–female social interaction. **(A)** Genotype and OXT treatment do not affect the amount of time spent in locomotion, exploration, self-grooming, or social investigation during the test. **(B)** *Oprm1*^−/−^ mice emit a reduced number of ultrasound vocalizations during the test in comparison with WT mice, and this deficit is completely rescued by OXT treatment. Data expressed as mean and SEM of eight to nine animals. *p < 0.05, **p < 0.01 compared to *Oprm1*^−/−^ + veh group.

To evaluate the pro-social effects of OXT, we tested sexually naive males during courtship, before the initiation of sexual behavior, during the first minutes of interaction with a female. The behavioral profile of males interacting with females is shown in Figure 5. The MANOVA indicated no significant effect of main factors (genotype: λ = 0.90, *F*_(5/27)_ = 0.58, n.s.; treatment: λ = 0.89, *F*_(5/27)_ = 0.61, n.s.), but a significant genotype × treatment interaction (λ = 0.62, *F*_(5/27)_ = 3.56, *p* < 0.05), suggesting that OXT had different effect on mice behavioral profile according to the genotype. Univariate results confirmed significant genotype × treatment effect for locomotion [*F*_(1/31)_ = 5.01, *p* < 0.05], social investigation [*F*_(1/31)_ = 4.22, *p* < 0.05], and USVs [*F*_(1/31)_ = 15.29, *p* < 0.001]. *Post hoc* analysis showed a significant difference at basal conditions between *Oprm1*^−/−^ and WT mice only in ultrasonic vocalizations (*Oprm1*^−/−^ + Veh vs. WT + Veh: *p* < 0.01), and an increase in the number of USVs due to OXT treatment, selectively in the *Oprm1*^−/−^ line (*Oprm1*^−/−^ + Veh vs. *Oprm1*^−/−^ + OXT: *p* = 0.01). By contrast, OXT did not to significantly modify WT males’ vocalizations (*p* = 0.13, n.s.) (Figure 5B). The deficit shown by *Oprm1*^−/−^ mice in USVs emission in response to social cues does not depend on a general impairment in their capacity to emit high levels of ultrasonic calls or in their sensitivity to olfactory stimuli, in infancy (16) as well as in adulthood (data presented here), but suggests lower motivation to interact with conspecifics.

The low impact of OXT on mutant males’ general social behavior (Figure 5A) can be related to the role of their mutant (untreated) females that may prefer to avoid their partner. In this respect, the USVs test will result more susceptible to treatment, as it only relies on the male behavior, independently on the female receptivity. Adult male mice emit USVs in the presence of female conspecifics and these vocalizations, promoting proximity, facilitate close social interactions, and sexual behavior. The advantage of testing males in this context stands in the consistent amount of ultrasonic calls that characterizes their first approach to the partner, which is independent from female’s receptivity and closely associated to the male’s motivational state (42–44). As a matter of fact, males emit USVs also when exposed to female’s cage bedding or urine (47). When in close proximity, mice can acquire information on the partner’s individual characteristics (e.g. sex, rank, sexual experience, and estrous condition) modulating their social behavior, and facilitating or avoiding social interactions. Moreover, the lack of strong deficits during sexual encounters in this mutant line confirms the entirety of their reproductive capabilities. Finally, the absence of significant effects of the intranasal OXT treatment in control male mice is in line with the Huang et al. study, showing only little changes in social investigation toward a female partner in C57BL/6J mice (34).

## Discussion

The aim of this study was to investigate, in *Oprm1*^−/−^ mice, the altered molecular mechanisms underlying the well documented deficits in social behavior presented by this animal model (16–19). In particular, we focused on alterations in the expression of brain OXTRs because they are the target of intranasally administered OXT, a promising new treatment for social deficits in autism (35, 48, 49). Our data show an OXTR up-regulation in specific brain regions of MOR-null mice, such as AONm, CeA, MeA, and NAcc. All these regions are involved in the regulation of social behavior, but, in our study, not all regions involved in social behavior that express OXTR were found to up-regulate the receptor. Furthermore, no correspondence between a high expression level of the OXTR and its up-regulation was observed. In particular: (1) regions that display very high levels of OXTR such as LS and BNST did not show any OXTR up-regulation and (2) of the regions where we observed OXTR up-regulation, three (AON, MeA, and CeA) have a high level of OXTR expression, and one (NAcc) has a medium-low level. Interestingly, a region-selective OXTR up-regulation was also observed in a recent paper in which OXTR expression levels were analyzed in the mouse brain after morphine treatment and withdrawal (50). However, the regions found in the aforementioned pharmacological manipulation study did not overlap with those described in the present study, an observation consistent with multiple patterns of receptor modulation linked to specific genetic, developmental, and pharmacological conditions. AONm, MeA, CeA, and NAcc are, nevertheless, all characterized by a medium-high MOR expression in WT mice (Table 1), suggesting a local interplay between the two receptor systems, which may involve neuroanatomical, cellular, or even subcellular interactions.

An open question regards, which is the neurobiological mechanism at the basis of the OXTR up-regulation. It is difficult to answer to this question as the OXT content and capability of OXT release in the hypothalamus of *Oprm1*^−/−^ mice has not been evaluated so far. If reduced, an up-regulation of the OXTR in target sites of the neuropeptide’s action may represent a compensatory mechanism to normalize the neuronal responses to reduced levels of secreted OXT. Becker et al. (19) actually found a reduced level of OXT transcript in *Oprm1*^−/−^ mice, but this impairment was restricted to the NAcc, leaving the overall levels of OXT undetermined. A second possibility implies direct molecular interactions between OXTR and MOR. G-protein coupled receptors such as OXTR and MOR, can form homo- (with another molecule of the same receptor) or hetero- (with receptors from other families) dimers and it has been shown that this physical association can modulate receptor binding and function (51). Interestingly, in mice lacking the alpha2A-adrenergic receptor, morphine has a reduced analgesic effect, suggesting that alterations in the noradrenergic system induce alterations in the opioid system (52). Molecular dimerization of MOR and alpha2A-adrenergic receptor has been demonstrated in both cell lines and primary neuronal cultures (53) and it has been shown that, in MOR–alpha2A-adrenergic receptor dimers, the activation of MOR by morphine inhibits the adjacent alpha2A-receptor by blocking its ability to activate the G-proteins even in the presence of noradrenaline (54). If dimeric interactions occur between MOR and OXTR, the lack of MOR may result in specific alterations of OXTR pharmacology in the brain regions in which the two receptors physically interact. Even if only speculative at present, this hypothesis will deserve further investigation.

A second question is: what are the neuroanatomical basis of the specific OXTR up-regulation in NAcc, CeA, MeA, and AONm of MOR-null mice?

As the NAcc is involved in the processing of social motivation and reward, it is not surprising that alterations in OXTR levels in NAcc have been found to be linked to altered social behaviors. First of all, natural variability in OXTR expression in the NAcc has been associated to social organization and mating behavior in voles, being the level of OXTR in the NAcc higher in monogamous voles displaying greater maternal care (55). Many other studies associated OXTR density in the NAcc to the degree of maternal and affiliative behavior and partner preference formation (56–60). Regardless of whether differences in OXTR expression are intrinsic (due to individual or species-related variability) (55, 57, 58), exogenously induced by viral-mediated over expression (35, 59, 60), or physiologically caused by labor and lactation (56), an higher OXTR level in these brain regions correlates with more pronounced social behavior. On the contrary, reduced levels of OXTR binding in the NAcc were observed in voles after paternal deprivation (61). At the neurophysiological level, presynaptic OXTRs have been recently shown to be required for social reward in the NAcc (33). In particular, OXTRs located on axonal terminals of serotoninergic projections coming from the dorsal raphe have been found to elicit Long-Term Depression (LTD) in medium spiny neurons by activation of serotoninergic 5HT1B receptors (33). NAcc neurons express high levels or MOR, but the neuronal populations that mediate all the distinct opiate effects remain elusive; recently, a subpopulation of striatal direct-pathway neurons was shown to support opiate reward-driven behaviors (62), and specific “hotspots” involved in the modulation of hedonic “liking” responses in which opioid receptors might function as hedonic enhancers have been identified (63). Furthermore, activation of MORs within the dorsal striatum appears to be critical to partner preference formation by generating socially motivated behavioral responses (9). OXTR may thus be postulated to act on specific MOR positive NAcc subpopulation(s) to regulate specific aspects or components of social reward, possibly via a presynaptic mechanism similar to that observed for the OXT induced LTP at 5HT1B receptors.

The different nuclei of the amygdala are responsible for the processing of emotional content of both positive and negative experiences. The CeA, as part of the brain’s reward circuitry, is important in processing positive emotions and, particularly, in the learning process of stimulus–reward association (64, 65). Moreover, the CeA is strongly implicated in the expression of fear. In mice, a neural circuit within the CeA that gates fear responses activating active strategies of risk assessment or passive expression of fear (freezing) has been described and, in this circuit, the OXTR was found to be a key player (66). Interestingly, as it has been observed that excessively aggressive animals show highest OXTR binding in the CeA (67), it is tempting to speculate that the increased aggressive behavior observed in *Oprm1*^−/−^ mice (19) may be linked to the selective up-regulation of OXTR in the CeA. The MeA is highly activated during social encounters and recognition, thus representing a critical neuroanatomical substrate of social cognition. The recognition of conspecifics is an initial and crucial condition for the establishment of social and sexual behavior. Odor, scent, and pheromones mediate sexual and competitive interactions, and are important in individual and kin recognition and mate selection. In rodents, odor signals are processed by two systems: the main olfactory pathways and the vomeronasal pathway, both directly and indirectly projecting to the MeA, a key region for the processing of chemosensory information (68). OXT-deficient mice are not able to recognize a previously encountered, familiar conspecific, and this social recognition behavior can be restored with a direct microinjection of OXT into the MeA of OXT KO mice, indicating that OXT in the MeA is both necessary and sufficient for social recognition (69, 70). Furthermore, male social interest correlated positively with OXTR in the MeA, while female social interest correlated negatively with OXTR expression in the same region (71). Finally, it has recently been shown that OXT-mediated LTD in the AOB–MeA pathway is directly involved in long-term social recognition memory formation (72). The up-regulation of OXTR in MeA may thus be involved in the pharmacological rescue of intranasal OXT on the social interaction test used in our study.

The up-regulation of OXTR in AONm of *Oprm1*^−/−^ remains of difficult interpretation. The *pars medialis* of the AON can be distinguished from the rest of the AON by cytoarchitectural features and projection patterns (73, 74), suggesting a different role than a station along the main olfactory and the vomeronasal pathways, but the role of OXTR in this region is still undefined.

Finally, what is the pathophysiological meaning of the OXTR up-regulation in *Oprm1*^−/−^ mice? As *Oprm1*^−/−^ mice do have severe defects in social interaction and social memory (19), the up-regulation of OXTR binding sites observed in *Oprm1*^−/−^ mice is apparently unable to normalize social cognition in these animals. Nevertheless, the up-regulation of OXTR may contribute to the rescue effects observed with intranasal OXT in MOR-null animals, as these KO animals completely recovered from communication deficits during the first minutes of social interaction, not differing from wild type animals anymore. Indeed, the present study confirms the efficacy of OXT treatment in reverting social deficits in a mouse model in which the oxytocinergic system underwent a compensatory modulation. These findings are of high relevance in the autism field as they further validate the use of OXT in ASD regardless of the primary etiopathogenetic cause(s) but solely on the basis of the social endophenotype. To fully understand the role of OXTR compensatory roles, it will be very interesting to investigate in detail the time-course of OXTR up-regulation in crucial periods of prenatal and post-natal life, including birth, weaning, and sexual maturity. Similarly, we only investigated male animals, but, due to the sexual dimorphism of OXTR expression in different species and brain areas (75, 76), similar analysis in female animals is warranted, as this may have a profound impact on the design of therapeutic schemes and doses.

In conclusion, the OXT system may act as a downstream effector toward social behaviors, compensating and/or bypassing genetic alterations in upstream circuits that produce social deficits. Such a hypothesis would justify the use of this neuropeptide in a wide variety of human conditions characterized by defects in social cognition and/or reward, such as ASDs, upon demonstration of OXT/OXTR modification(s) in the brain of affected individuals. Moreover, the results presented here further corroborate the hypothesis that alterations in the OXTergic system are involved in the pathophysiology of autism and that administration of OXT to such altered system might restore a balance in the neural circuitries involved in social behavior.

## Author Contributors

Valentina Gigliucci: performed experiments (autoradiography, Nissl staining, and AChE) and data analysis; wrote the manuscript. Marianna Leonzino: performed data analysis and wrote the manuscript. Marta Busnelli: performed experiments (autoradiography and AChE staining) and data analysis. Alessandra Luchetti: performed experiments (pharmacological treatment and behavioral tests). Viola Stella Palladino: performed experiments (animal genotyping and behavioral tests). Francesca R. D’Amato: conceived and supervised behavior experiments and wrote the manuscript. Bice Chini: conceived and supervised the study and wrote the manuscript.

## Conflict of Interest Statement

The authors declare that the research was conducted in the absence of any commercial or financial relationships that could be construed as a potential conflict of interest.
